# Impact of pharmaceutical promotion on prescribing decisions of general practitioners in Eastern Turkey

**DOI:** 10.1186/1471-2458-7-122

**Published:** 2007-06-25

**Authors:** Serhat Vancelik, Nazim E Beyhun, Hamit Acemoglu, Oksan Calikoglu

**Affiliations:** 1 Department of Public Health in Ataturk University, School of Medicine, Erzurum, Turkey; 2 Department of Medical Education in Ataturk University, School of Medicine, Erzurum, Turkey; 3 Ataturk Universitesi Tip Fakultesi Halk Sagligi ABD, 25240, Erzurum, Turkey; 4 Ataturk Universitesi Tip Fakultesi, Tip Egitimi ABD, 25240, Erzurum, Turkey

## Abstract

**Background:**

Commercial sources of information are known to have greater influence than scientific sources on general practitioners' (GPs) prescribing behavior in under developed and developing countries. The study aimed to determine the self-reported impact of pharmaceutical promotion on the decision-making process of prescription of GPs in Eastern Turkey.

**Methods:**

A cross-sectional, exploratory survey was performed among 152 GPs working in the primary health centers and hospitals in Erzurum province of Eastern Turkey in 2006. A self-administered structured questionnaire was used. The questionnaire included questions regarding sociodemographics, number of patients per day, time per patient, frequency of sales representative visits to GPs, participation of GPs in training courses on prescribing (in-service training, drug companies), factors affecting prescribing decision, reference sources concerning prescribing and self-reported and self-rated effect of the activities of sales representatives on GPs prescribing decisions.

**Results:**

Of 152 subjects, 53.3% were male and 65.8% were working at primary health care centers, respectively. Mean patient per day was 58.3 ± 28.8 patients per GP. For majority of the GPs (73.7%), the most frequent resource used in case of any problems in prescribing process was drug guides of pharmaceutical companies. According to self-report of the GPs, their prescribing decisions were affected by participation in any training activity of drug companies, frequent visits by sales representatives, high number of patient examinations per day and low year of practice (p < 0.05 for all).

**Conclusion:**

The results of this study suggest that for the majority of the GPs, primary reference sources concerning prescribing was commercial information provided by sales representatives of pharmaceutical companies, which were reported to be highly influential on their decision-making process of prescribing by GPs. Since this study was based on self-report, the influence reported by the GPs may have been underestimated.

## Background

Drug expenditure has been one of the main concerns of health care managers; thus, its containment is one of the primary goals of health care authorities. Therefore, identifying prescribing-associated factors is of paramount interest from health, as much as social and economic standpoints [1-4]. The effects of various factors on prescribing decisions have been considered in many studies [5-7]. The physician's age, training, environment, and health-care demand have been quoted as explanatory factors for prescribing behavior.

As Prosser et al mentioned factors like doctor characteristics, hospital consultants, the pharmaceutical industry and patient characteristics lie behind the prescribing decisions among general practitioners (GPs) [8]. In our study, some factors which were thought to be potentially related with prescribing decisions of GPs were considered. In this context, the number of patients examined per day may be an influential factor on prescribing decisions because the frequency of visits by sales representatives (SRs) to the physicians examining high number of patients per day may tend to be higher. Therefore, high number of patients examined per day means shorter time per patient. Furthermore, shorter time per patient may affect rational decision-making process regarding prescription. Work site is considered as another possible influential factor because daily burden of GPs with regard to number of patients per day in primary health care settings is relatively higher than that in the hospitals in Turkey. As mentioned above, patient per day may be an important parameter to determine the effect of pharmaceutical promotion on GPs. Year of practice of GPs may also affect prescribing decisions. Higher number of practice years may indicate more experience in prescribing, and thus, the influence of SRs on experienced GPs may be lower.

Commercial sources of information are known to have a greater influence than scientific sources on general practitioners' prescribing behavior in under developed and developing countries. Twenty years ago, Avorn et al found that although physicians believed that drug advertisements and pharmaceutical representatives had a minimal effect on their prescribing behavior, they held advertising oriented beliefs about the efficacy of drugs such as cerebral vasodilators and dextropropoxyphene [9]. Recently, in a survey of 200 general practitioners and 230 hospital based doctors, the information on the latest new drug prescribed was derived from pharmaceutical representatives in 42% of cases [10].

A systematic review also found that meetings with representatives were associated with requests by physicians for promoted drugs to be added to the hospital formulary, requiring changes in prescribing practice with increased prescribing costs and less rational prescribing [11]. Therefore, interactions between physicians and drug companies raise scientific and ethical questions.

We considered that the pharmaceutical promotion had a significant effect on prescribing decisions. Patient per day, work setting, year of practice, gender, frequency of sales representatives (SRs) visits, participation in training sessions were expected to be the determinants of the effect of pharmaceutical promotion. The study aimed to determine the self-reported impact of promotional activities by sales representatives of pharmaceutical companies on the decision-making process of drug prescription ordered by the general practitioners (GPs) working in Eastern Turkey.

## Methods

### Study site

This descriptive and exploratory study was conducted with the participation of general practitioners (GPs) who were working in Erzurum, a metropolitan city in Eastern Turkey. The GPs who were working at 12 primary health care centers, 2 city hospitals, 1 respiratory diseases hospital, and 1 gynecology-obstetrics hospital were included in the study population.

### Study design and subjects

Involvement of all the GPs in the study population was aimed. Total number of GPs working in Erzurum Province (including rural region) was 157. However, 2 of GPs (1.3%) refused to participate, and the remaining three GPs (1.9%) were absent due to their illness. The participation rate was 96.8% (152/157).

The data was collected during January-February 2006 by researchers. All the GPs were informed on the study objective and the data was collected via a self-administered structured questionnaire. The questionnaires were filled out by the GPs and the researchers were present on the site while the GPs were filling out the questionnaire. If the GPs were not able to fill out the questionnaires because of the heavy workload or were not available, they were visited a second and third time. Additionally, Regional Health Directorate of Erzurum Province supported the study and encouraged the GPs to participate.

### Questionnaire

The questionnaire included questions regarding sociodemographics, number of patients examined per day, time per patient, frequency of sales representative visits to GPs, participation of GPs in training courses on prescribing (in-service training, drug companies), factors affecting prescribing decision, reference sources concerning prescribing and self-reported influence of activities of sales representatives on prescribing decision.

Mean patient per day was determined by using the files of the last 3 months before the study. In the light of the recommendations by World Health Organization (WHO), 10 patient examinations of each GP were observed by a single researcher (OC) in order to determine the examination time per patient [12].

There were 5 questions to measure the self-reported and self-rated effect of pharmaceutical promotion on prescribing in the questionnaire (See questions in Table 1). Regarding Q1. the answers of 1–2, 3–4 and 5–6 were classified as high, moderate and low, respectively. For Q2., the answers of 1–2, 3–4 and 5–6 were also classified as frequently, sometimes and rarely, respectively. In the analysis of Q5, the answers of "yes/always" and "yes/sometimes" were combined and compared to the answer "no". The frequency of visits of SR were also asked to the GPs as more than 1 per week – more than 1 per month and less frequent. It was further classified as more than 1 per month and less frequent in the analysis.

**Table 1:** Questions about prescribing decision and its determinants.

**Q1**. Please rate the factors below according to the level of the effect on your prescribing decision in the order of 1 (low) to 6 (high).
- Pharmacology courses during formal medical education.
- Activities of sales representatives
- Observation of prescribing during clinical practice in medical school
- Post-graduate in-service training by public sector
- Consultations with other physicians
- Self-reading after graduation
**Q2**. Please rate the references that you consulted according to frequeny of use in case of any problem in prescribing, in the order of 1 (frequently) to 6 (rarely).
- Drug guides of pharmaceutical companies
- Documents of pharmaceutical companies other than drug guides (brochures etc...)
- Medical text books
- Academic journals
- Consultation with a specialist doctor
- Consultation with other GPs

**Q3**. Have you been involved any kind of postgraduate training programme on prescribing?
- Yes
- No

**Q4**. If yes, what kind of programmes were they?
- Training programmes of pharmaceutical companies
- In-service training provided by public sector
- MsC/PhD programme
- Pharmacology courses during medical faculty

**Q5**. Do the activities of sales representatives of drug companies affect your prescribing decision?
- Yes/always
- Yes/sometimes
- No

### Statistical Analysis

Statistical analyses were performed using the Epi Info (version 6.04) developed by Centers for Disease Control and Prevention. Determinants of self-reported effect were analysed with the Pearson Chi-Square analysis. *P value under *0.05 was considered to be significant.

### Ethics and consent

The study was approved by the Ethical Committee of General Health Directorate of Erzurum Province. As this study was on human subjects, Helsinki Declaration was signed by all of the authors. Written informed consents of all the GPs were collected by the researchers.

## Results

Characteristics of the study population have been shown in Table 2. Of the subjects, 53.3% were male, and 65.8% were working at primary health care centers. The mean year of practice was 6.3 ± 3.1 years (median = 6 years, min-max = 1–14 years). Of the GPs, 24.3% had 1–3 years, and 17.8% had more than 10 years of practice. The mean patient per day was 58.3 ± 28.8 (median = 50, min-max = 18–103) per GP. Of the GPs, 47.4 and 21.1% examined more than 60 and 90 patients a day, respectively. The mean time of examination per patient was 8.2 ± 4.7 (median = 6.4, min-max= 3.4 – 16.7) minutes. While 75.6% of the GPs had participated in training programs of pharmaceutical companies, 28.2% had gone into in-service training provided by public sector on prescribing. In addition to this, 72.3% of the physicians were visited by SRs more than once a month. Of the GPs, 61.2% reported that their prescribing desicions were always affected by SRs activities.

**Table 2:** Characteristics of the study population

	**Frequency**	
**Characteristics (N = 152)**	**n**	**%**

**Gender**		
Male	81	53.3
Female	71	46.7

**Years of practice**		
1–3	37	24.3
4–6	50	32.9
7–9	38	25.0
10 and above	27	17.8

**Work Setting**		
Primary health care center	100	65.8
Hospital	52	34.2

**Number of patients per day**		
30 and under	23	15.1
30 – 59	57	37.5
60 – 89	40	26.3
90 and over	32	21.1

**Time per patient**		
<5 min	19	12.5
5–10	109	71.7
>10	24	15.8

**Participation in any training programmes of drug companies**		
Yes	117	77.0
No	35	23.0

**Participation in in-service training**		
Yes	43	28.2
No	109	71.8

**Frequency of SR visits to GPs**		
More than once a month	110	72.3
Less frequently	42	27.7

**Self-reported effect of SRs activities on prescribing decision**		
Yes/always	93	61.2
Yes/sometimes	45	29.6
Never	14	9.2

Factors, which are rated by GPs, that affect their prescribing decisions and the frequency of using reference sources in case of any problems in their prescribing process have been shown in Table 3. The most frequent resources used in case of any problems in prescribing process were drug guides of pharmaceutical companies (73.7%), medical books (48.7%) and the documents of pharmaceutical companies other than drug guides (33.6%). Academic publications and consultation made with other GPs were the least frequently used resources. The GPs endorsed that self reading after graduation (50.7%) and activities of pharmaceutical representatives (40.7%) had high effect on their prescribing decisions. Pharmacology courses at medical school (49.4%) and post-graduate in-service training provided by public sector (42.1%) had low effect according to the GPs statements. Of the GPs, 77.0% went into training on prescribing, 53.0% of these GPs only participated in educational activities of pharmaceutical companies. Remaining 47.0%, participated in a training programme provided by both the pharmaceutical companies and other sources (in-service training provided by public sector, courses of universities).

**Table 3:** Factors, which are rated by GPs, that affect their prescribing decisions and the frequency of using reference sources in case of any problems in prescribing.

	**Level of effect**		
	
	**High**	**Moderate**	**Low**
**Factors**	**%**	**%**	**%**
Self reading after graduation	50.7	31.5	17.8
Activities of sales representatives	40.1	30.3	29.6
Observation of prescribing during clinical practice in medical school	37.5	39.5	23.0
Pharmacology courses during formal medical education.	34.2	16.4	49.4
Consultation with other physicians	24.3	39.5	36.2
Post-graduate in-service training provided by public sector	13.2	44.7	42.1

	**Frequency**		
	
	**Frequently**	**Sometimes**	**Rarely**
**Reference resources**	**%**	**%**	**%**

Drug guides of pharmaceutical companies	73.7	19.7	6.6
Medical text boks	48.7	38.1	13.2
The documents of pharmaceutical companies**	33.6	32.8	33.6
Consultation with a specialist doctor	19.1	41.4	39.5
Consultation with other GPs	15.8	32.2	52.0
Academic journals	9.2	35.5	55.3

* Row %

** Educational documents other than drug guides (brochures etc...)

Determinants of the self-reported effect of SR activities on prescribing decisions of GPs have been shown in Table 4. The percentage of the affected GPs who had participated in training courses of pharmaceutical companies was significantly higher than the percentage of the affected GPs who did not participate in any training of companies (94.0% vs. 80.0%, p = 0.019). The proportion of the affected GPs working at primary health care centers was significantly higher than that of the affected GPs working at hospitals (95.0% vs. 82.7%, p = 0.018). The percentage of the affected GPs whose year of practice was 5 years and under was significantly higher than the percentage of affected GPs who were more experienced (96.0% vs 85.7%, p = 0.047). The proportion of the affected GPs who examined 60 or more patients a day was significantly higher compared to the affected GPs with a daily patient burden fewer than 60 (95.8% vs. 86.3%, p = 0.041). The percentage of the affected GPs who were visited by SRs more than once a month were significantly higher than affected GPS who were less frequently visited (95.4% vs. 78.5%, p = 0.003).

**Table 4:** Determinants of the self-reported effect of the SRs' activities on prescribing decisions of the GPs.

	**Always or sometimes affected**		**Never affected**		
	
**Determinants (N = 152)**	**N**	%**	**N**	%**	**P**
**Gender**					
Male	75	92.6	6	7.4	
Female	63	88.7	8	11.3	

**Participation in any training programmes of drug companies**					*
Yes	110	94.0	7	6.0	
No	28	80.0	7	20.0	

**Participation in in-service training**					
Yes	31	93.9	12	6.1	
No	107	89.9	2	10.1	

**Work Setting**					*
Primary health center	95	95.0	5	5.0	
Hospital	43	82.7	9	17.3	

**Frequency of SR visits to GPs**					*
More than 1 per month	105	95.4	5	4.6	
Less frequently	33	78.5	9	21.5	

**Years of practice**					*
5 and under	72	96.0	3	4.0	
More than 5	66	85.7	11	14.3	

**Patient per day**					*
<60	69	86.3	11	13.7	
60 and more	69	95.8	3	4.2	

* Statistically significant, p values (exact sig. 2-sided) under 0.05

** Row percent

**Figure 1:**
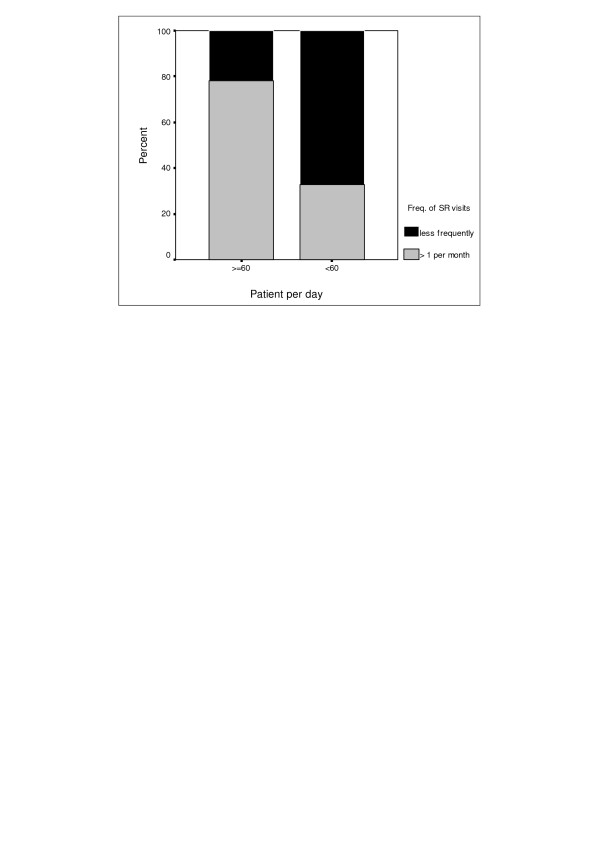
Frequency of SR visits to GPs according to number of patient examinations per day.

Frequency of SRs visits to the GPs according to the number of patient examination per day has been shown in Figure 1. If the GPs examined 60 and more patients per day, the frequency of SR visits was more frequent than once a month and the rate was significantly higher than the visits made to the GPs who examined fewer than 60 patients per day (90.3% vs. 56.3%, p = 0.002). Additionally, the physicians who examined 60 and more patients a day were involved in training courses of pharmaceutical companies more frequently than the physicians who examined fewer than 60 patients a day (97.2% vs. 57.5%, p = 0.003).

## Discussion

This cross-sectional and exploratory study was performed with the participation of 152 GPs working at primary health centers and hospitals in order to find the effect of pharmaceutical promotion on prescribing decisions. According to the self report of the GPs, their prescribing decisions were always or sometimes affected significantly if they had participated in any educational activity of pharmaceutical companies and if they were working in primary health care centers, respectively (p < 0.05 for both). The self-reported effect of pharmaceutical promotion on the prescribing decisions of GPs was significantly higher if the GPs were visited by SRs frequently, i.e. more than once a month; examining 60 or more patients per day, and year of practice was 5 years and under, respectively (p < 0.05 for all).

The rate of SRs visits to GPs who had a high burden of patient per day was higher. These GPs also reported high influence by the activities of SRs. These findings may be the key factors to show the effect of pharmaceutical companies on prescribing decisions of GPs. Prosser et al. stated that the characteristics and working conditions of the GPs were generally underestimated while investigating the pathways of the effect of pharmaceutical promotion on GPs [8]. Like Prosser et al, we found that if the GPs were working at primary health care centers and had an experience less than 5 years after graduation, they reported higher influence on their prescribing decision. Additionally, the GPs under heavy burden of high number of patients per day could provide limited time per patient, which may affect the rational prescribing decision in a negative manner. The self-rated effect of pharmaceutical promotion on prescribing decisions was lower for experienced GPs. Total year of practice was found to be another factor to have an influence on the pathway of prescribing decision.

Similar to our study, many previous studies also determined that GPs were affected by promotions of pharmaceutical companies [13-19]. According to several primary care physicians, detachment within the health care system, especially in the traditional primary care model, is exploited by pharmaceutical companies to create personal links with the physicians [20]. This may have an effect on a more positive perception of the quality of the information provided by sales representatives while affecting the physician's prescribing behavior. In a 2001 survey of random sample of U.S physicians, 92% of the physicians received free drug samples from companies; 61% received meals, tickets to entertainment events, or free travel; 13% received financial or other in-kind benefits [21]. These incentives may be dominant for GPs who prescribed high number of drugs. In this study, we noticed that the frequency of the visits to GPs performed by SRs was higher for the GPs with high number of patients per day (Figure 1). This may suggest the presence of the effect of pharmaceutical promotion on GPs.

Of the GPs, 77.0% received education on prescribing. Whereas 53.0% of these only participated in training courses of pharmaceutical companies, the remaining 47.0% received education from both the drug companies and other sources (in-service training, course of universities). It was found that there was a lack of postgraduate medical education provided by public sector (in-service training) in GPs in Eastern Turkey. Thus, participating in educational courses of pharmaceutical companies was common among the GPs. All of the GPs, who had been involved in any pharmaceutical education activity, had received at least one suchlike training programme from drug companies. Patient per day ratio per GP was also significant, indicating the involvement of GPs in training programmes of pharmaceutical companies. This is possibly due to the higher frequent visit of SRs to GPs with higher number of patients per day. Previous studies also determined that most physicians allocate more hours to receiving SRs than to attending updating courses [20,22]. According to various authors, commercial information makes up for the lack of training in health care services, and this is even more common in developing countries where the drug industry influence is greater[23,24]. However, postgraduate medical education should not be completely dependent on the initiative of pharmaceutical companies, a well-known issue worldwide [25]. For example, the pharmaceutical industry is also the main provider of information to physicians in Spain [23]. The quality and content of formal pharmacology education during medical faculties is another factor that can directly affect the formation of prescribing decision and the attitudes of GPs towards the relations between doctors and pharmaceutical companies. Critics argue that basic pharmacology rather than problem solving and practical application or audit is overemphasized during medical training in developing countries, and largely responsible for establishing poor prescription habits that subsequently prove difficult to change [26-28].

In our study, drug guides of pharmaceutical companies, medical books, and the documents of pharmaceutical companies other than drug guides were the most commonly used reference resources in case of any problems in prescribing. The most common reference source used by 73.7% of the GPs in this study was drug guides of pharmaceutical companies. It has been reported that 86% of the GPs in Tunisia mainly use drug guides when any prescribing problems arise, and nearly 30.0% do not refer to any medical publications [29]. Drug guides prepared by pharmaceutical companies may have a negative effect on rational prescribing behavior of GPs. Nevertheless, various studies determined the great extent of the effect by the pharmaceutical industry on prescribing behavior of GPs [30,31]. In our study GPs reported that self-reading after graduation and pharmaceutical promotion were the leading factors that affected their prescribing decisions. As mentioned above, this finding also indicates the lack of in-service training provided by public sector. In addition to this, consultation between GPs and other specialists was quite inadequate. (Table 3). Heavy patient burden might play a role in these inadequate interpersonal consultations. Pharmaceutical companies may fulfill the gaps occurring because of limited communication between physicians.

The findings of this descriptive study were based on self-report of GPs about the effect of pharmaceutical promotion on their prescribing decision. The reliance of self-report is one of the main issues of the studies similar to ours. Blumenthal et al. noted that in a study of residents, it was found that 61% believed that they were not influenced by the marketing efforts of pharmaceutical companies, although only 16% were equally confident about their colleagues [21]. Carthy et al stated that GPs considered themselves as cautious and conservative prescribers. In this study, GPs also stated that they were not unduly influenced by the drug representatives [32]. Like Prosser and Avorn et al. and based on the findings of other studies above, there might be an underestimation of the effect of pharmaceutical promotion on the prescribing decision of the GPs in our study.

As this was a descriptive and exploratory study in a single province of Eastern Turkey, we aimed the enrollment of all the GPs in the area; therefore, we did not use any sampling method. The participation rate was relatively high (96.8%), and this was one of the strengths of our study. This might be due to the support of local health directorate in the enrollment and willingness of the GPs to participate. The official regulations governing the pharmaceutical promotion was arranged by law in Turkey in 2003 and since then it has been in effect. This law provided restrictive mandatory regulations to pharmaceutical promotion. However, the implementation of the regulation was not adequately monitored by Ministry of Health.

## Conclusion

This is a unique study, which was conducted in one of the less developed regions of a developing country, Turkey. The results of this study suggest that the promotional and educational courses of pharmaceutical companies were reported to be influential on their prescribing decisions by GPs. In addition to this, for the majority of the GPs, primary reference sources concerning prescribing was commercial information provided by sales representatives of pharmaceutical companies. All these results indicates a lack of formal continuing medical education and an adequate monitoring of prescribing behaviours provided by public sector.

## List of abbreviations

GP: General practitioner

SR: Sales representative

## Competing interests

The author(s) declare that they have no competing interests.

## Authors' contributions

All of the authors read and approved the final manuscript.

SV participated in the development of the protocol and analytic framework for the study, outcome assessment, GPs enrollment, and manuscript preparation.

NEB had primary responsibility for protocol development and contributed outcome assessment, preliminary data analysis, and writing the manuscript.

HA supervised the design and execution of the study, contributed to the final data analyses and manuscript preparation.

OC supervised the design and execution of the study, contributed to the final data analyses and GPs enrollment.

## Pre-publication history

The pre-publication history for this paper can be accessed here:


